# Management of chyluria using percutaneous thoracic duct stenting

**DOI:** 10.1186/s42155-022-00333-y

**Published:** 2022-10-19

**Authors:** Nguyen Ngoc Cuong, Le Tuan Linh, Thieu Thi Tra My, Tran Quoc Hoa, Hoang Long, Le Hoan, Masanori Inoue

**Affiliations:** 1 Diagnostic Imaging and Interventional Center, Hanoi Medical University Hospital, No1, Tonthattung, Dongda Hanoi, Vietnam; 2 Surgery of Urology Department, Hanoi Medical University Hospital, Hanoi Medical University, Hanoi, Vietnam; 3 Respiratory Department, Hanoi Medical University Hospital, Hanoi, Vietnam; 4 Keio University, Tokyo, Japan

**Keywords:** Chyluria, Lymphatic, Thoracic duct, Stenosis, Obstruction, Balloon, Stent

## Abstract

**Background:**

Thoracic duct stenosis or obstruction is one of the causes of chyluria. Although the diagnosis of chyluria is not difficult, treatment is still challenging. Although there have been no standard guidelines for the treatment of chyluria, interventional techniques now offer minimally invasive treatment options for chyluria such as interstitial lymphatic embolization, ductoplasty with balloon, or thoracic duct stenting.

**Case presentation:**

Here, we report a case of chyluria due to obstruction of the junction between the thoracic duct and subclavian vein in a 64 -year- old female patient. The patient was treated with balloon plasty for lymphovenous junction obstruction and interstitial lymphatic embolization for chyluria. However, chyluria was recurrent after 6 months so intranodal lymphangiography was performed. Anterograde thoracic duct was accessed through a transabdominal to the cisterna chyli which showed that the thoracic venous junction was re-obstruction. The patient was successfully treated by placing a uncovered drug-eluting stent with the size of 2.5 mm x 15 mm in length for resolving the thoracic occlusion.

**Conclusion:**

This report demonstrates the feasibility of using thoracic duct stenting in the treatment chyluria due to lymphovenous junction obstruction.

## Introduction

Chyluria is divided into parasitic and non-parasitic categories (Stainer et al. 2020). Non-parasitic chyluria is a rare condition and caused by: trauma, surgery, infections, malignancy, lymphatic malformation, radiation, urinary retention, congenital fistula between lymphatics and the urinary tract, pregnancy and stenosis of the thoracic duct (TD) (Stainer et al. 2020; Shah et al. 2020). There are no guidelines for the management of chyluria. The treatment approach depends on the etiology and the site of the lymphatic system damage; and mostly should be tailored on a case-by-case basis. Conservative treatment with a low-fat diet is the first step. Sclerotherapy using a ureteroscope has also been reported, but it is not a well-established treatment and its effectiveness is limited. As a result, not all patients respond well to these therapies (Lovrec Krstić et al. 2021). In the literature, some new alternative treatments were applied for the management of chyluria such as interstitial lymphatic embolization through percutaneous or retrograde thoracic duct access, and interstitial lymphatic embolization with balloon plasty (Gurevich et al. 2018; Hur et al. 2021; Nguyen et al. 2020). In this article, we would like to present a new interventional technique for a patient with chyluria associated with the lymphovenous junction (LVJ) obstruction.

## Case report

**Fig. 1:**
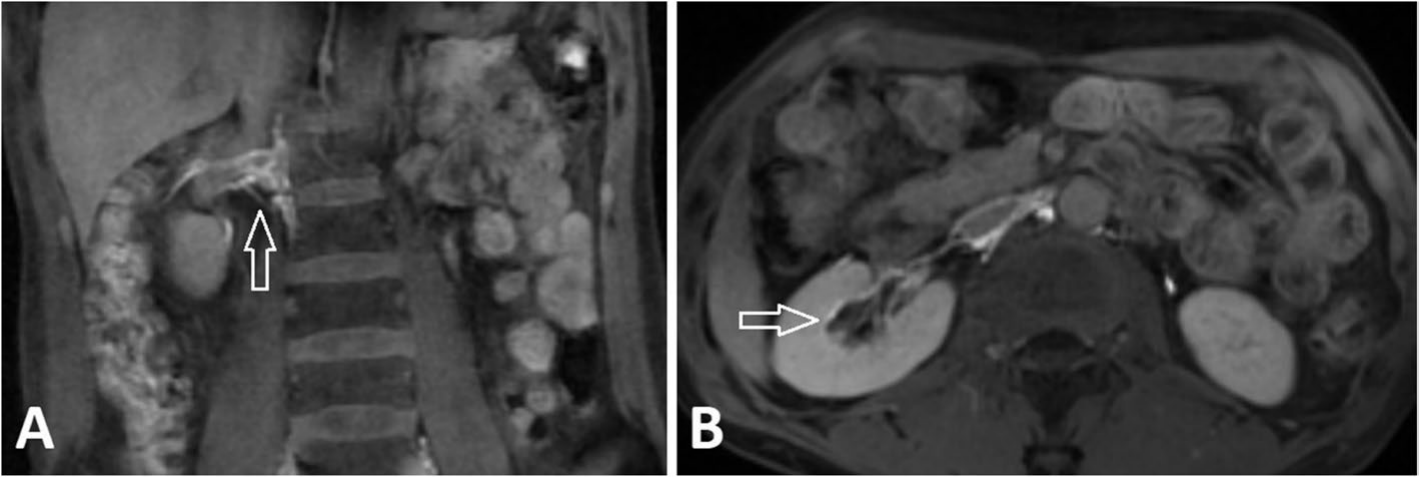
DCMRL showed dilated lymphatic vessels in the right renal pelvis (**A**) and the chylo calyceal fistula (**B**)

A 64 -year- old female patient has suffered from chyluria for 6 months. She has no history of parasite infection, no abdominal trauma, and no history of renal operation. Cystoscopy showed the milky chyle efflux from the right ureteric orifice suggesting that the origin of chyluria was from the right kidney. The patient underwent dynamic contrast-enhanced magnetic resonance lymphangiography (DCMRL). DCMRL visualized the thoracic duct and showed the presence of dilated lymphatic vessels in the right renal pelvis confirming the chylo calyceal fistula (Fig. 1).

**Fig. 2:**
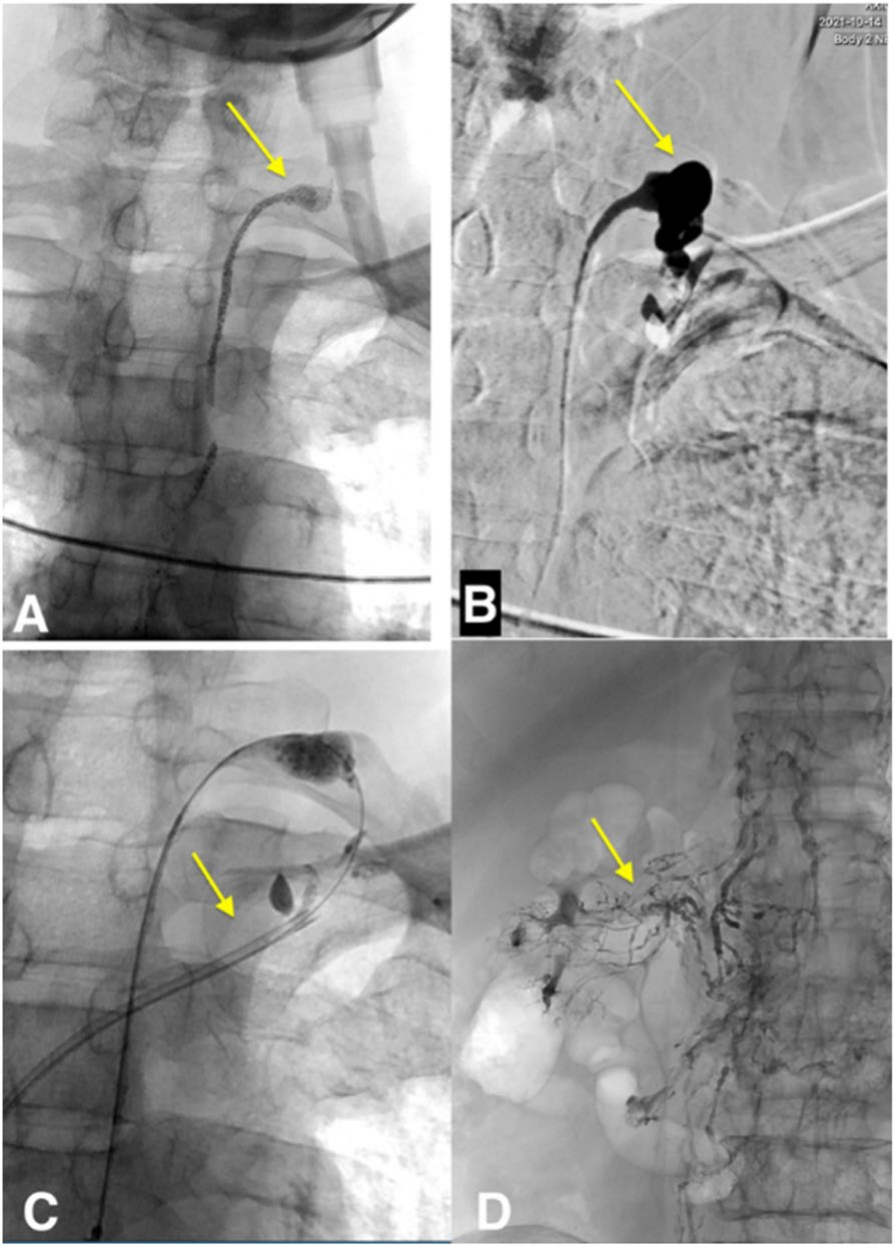
First intervention. **A** Lymphangiography revealed dilated the upper part of TD and remained the contrast in the TD (arrow). **B** injection of contrast into TD showed occlusion of the TD and dilatation at the junction (arrow). **C** The 0.014”-guidewire passed through the occlusion into superior vena cava then the femoral vein by a snare. A 6 French-guiding catheter was placed at the junction TD subclavian vein (arrow). **D** Right retroperitoneal lymphatic system communicates with renal collecting system and the contrast material presented in the kidney calyces (arrow), the interstitial lymphatic embolization was then performed

Bilateral inguinal intranodal lymphangiography was then performed. There was a stagnation of Lipiodol (Guerbet, France) in the lymphatic system and the cisterna chyli appeared after 50 minutes since the injection of contrast started. In addition, reflux of oil contrast into the right renal pelvis was also observed. On lymphography, the thoracic duct was also dilated and the contrast was ended at the TD venous junction without draining in the subclavian vein. Neck ultrasound and echocardiography revealed there was no neck tumor or central vein thrombosis. There were no anatomical abnormalities of the lymphatic system, LVJ as well as the left subclavian vein. The laboratory tests gave negative results with Wuchereria bancrofti, Toxocariasis, Taenia echinococcus. The diagnosis was made as chyluria from the right kidney due to an obstruction at the junction TD-subclavian vein. Because the guide wire (0.018” Terumo) could pass through the occlusion, the LVJ was dilated by balloon and then interstitial lymphatic embolization was done to occlude the lymphatic communication between retroperitoneal lymphatic vessels and the right renal pelvis as described in the literature (Gurevich et al. 2018; Kariya et al. 2019). We punctured the cisterna chyli by a 21-gauge-needle (chiba, Cook, USA) and then inserted a 0.018” guide wire into the thoracic duct. A 2.7-french-microcatheter (progreat, terumo, Japan) was advanced into the thoracic duct over the gude wire. By injecting the contrast into microcatheter in order to opacify the thoracic duct, we found that there was an occlusion at the TD venous junction (Fig. 2 A and B). The long guide wire (0.014”, 300 cm, transend soft tip, Boston Scientific, USA) was easily passed through the occlusion and then was advanced into the superior vena cava. From the right femoral vein, we advanced a snare into the superior vena cave. The snare caught the guide wire and then pulled the guide wire out of the sheath at the right femoral vein. TD venous junction was dilated at a pressure of 8 atm using a balloon 2.5 mm x 20 mm (Pantera LEO, Biotronik, Bulach, Switzerland) (Fig. 2 C). The interstitial lymphatic embolization was then performed with total of 2 ml mixture of N-butyl cyanoacrylate (NBCA) and lipiodol with the ratio of 1:5 (Fig. 2D) as describe in the literature during the inflation of the balloon at the junction TD vein (Gurevich et al. 2018).

**Fig. 3:**
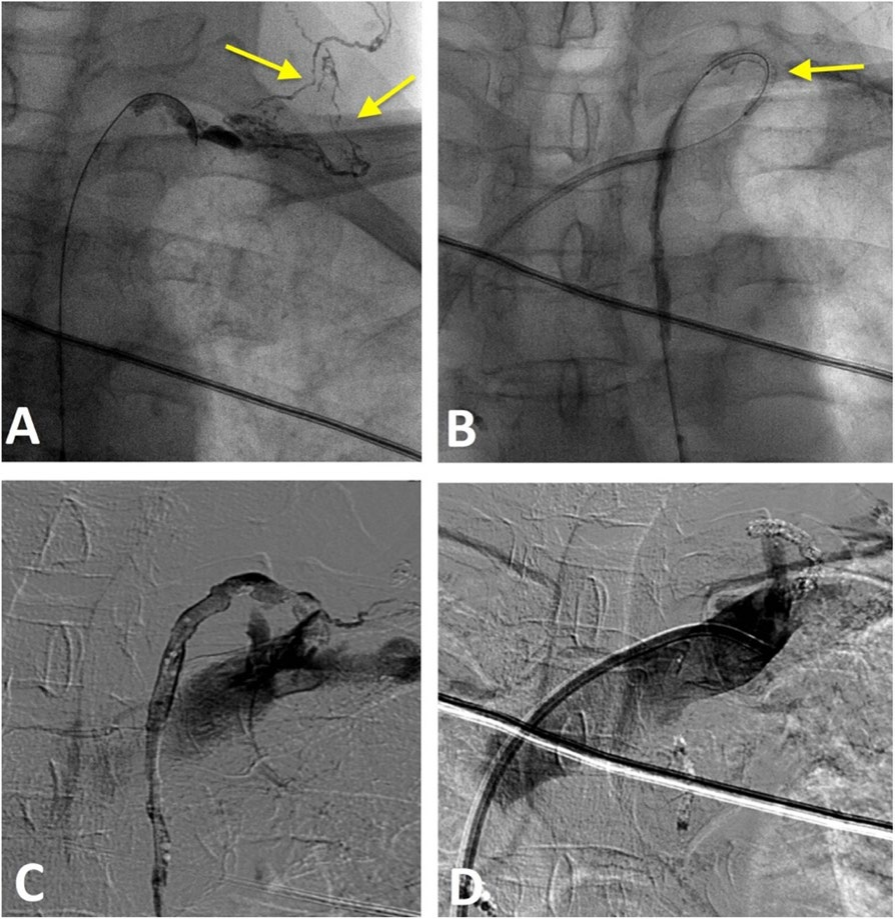
Second intervention. **A** TD lymphangiography showed re-occlusion of the junction TD vein and collateral circulation of lymphatic vessels in the left neck (arrows). **B** The stent was inflated in the LVJ by the balloon (arrow). **C** Injection of contrast after deploying the stent showed the flow into subclavian vein. **D** superior venogram showed that the contrast material in the left subclavian vein did not reflux to the TD

**Fig. 4:**
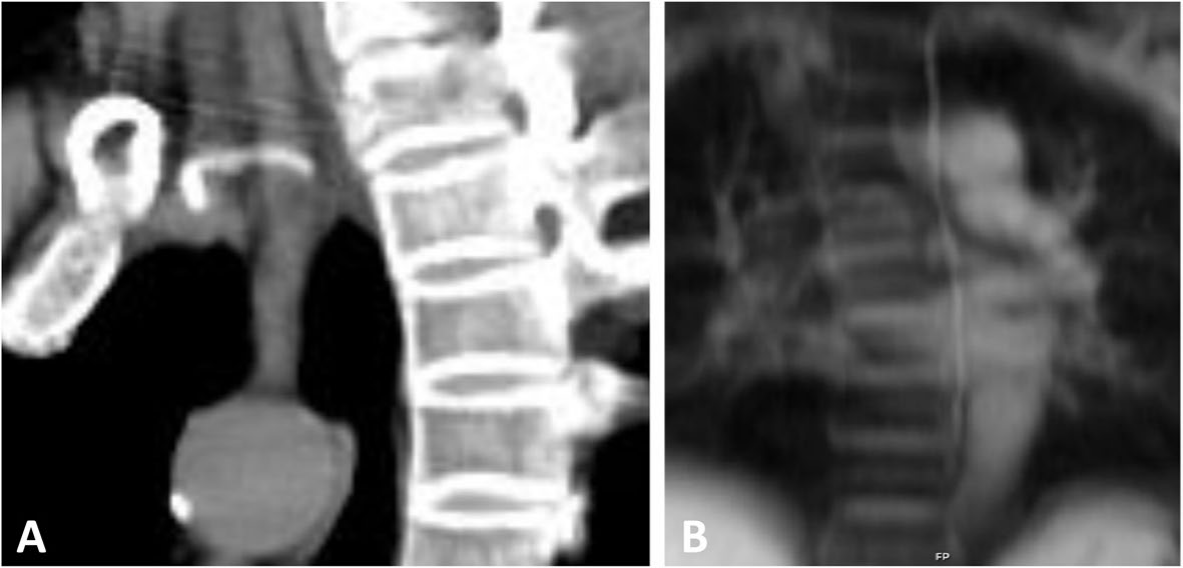
Follow up images. **A** CT scans 6 months post-stenting revealed the stent was in the right position. **B** DCMRL after one year showed the patency of thoracic duct

The patient recovered well with no longer chyluria; no complications were noticed. But 6 months after, the patient come back to our institution because of recurrent symptoms of chyluria. Cystoscopy showed the efflux of chyle on the right side, and DCMRL found again the afferent lymphatic vessels in the right kidney. We performed intranodal lymphangiography and found that there was re-obstruction of the TD venous junction. Dilated and tortuous right retroperitoneal lymphatics, right kidney lymphatics, and filling of kidney calyces like the previous lymphangiography was also revealed. Because re-obstruction occurred after balloon plasty 6 months, we planned to re-delate the junction by balloon and place a metallic stent. The stent we used was a coronary stent with the size of 2.5 mm x 15 mm in length (drug-eluting stent) because of the compatible size (Fig. 3). After the stent deployment, direct lymphangiography demonstrated contrast medium from the thoracic duct to the left subclavian vein without disruption or leakage. Venography of the left subclavian vein showed no reflux flow from the vein into the thoracic duct. Then, we intended to puncture the interstitial lymphatic vessels at the lumbar region, but all attempts failed. Therefore, no embolization of interstitial lymphatic vessels was performed. After procedure, the patient had transient hematuria but no chyluria. The patient had remained asymptomatic for 1 year later and still under following up. The computed tomography (CT) scan at the 6th month showed the stent fully opened and was in the correct position (Fig. 4 A) and DCMRL lymphangiography at the 12th month showed the patency of TD (Fig. 4B).

## Discussion

The TD can be injured via trauma, surgery, or congested by central venous occlusion (An et al. 2021; Chick et al. 2018). It may be caused by compression from outside. Other causes include lymphatic malformation, radiation, congenital abnormalities, and stenosis of the TD (Lovrec Krstić et al. 2021). Valvular insufficiency in the thoracic duct results in elevated pressure in the lymphatic circulation and backflow of lymphatic fluid into other major lymphatic branches. When the thoracic duct pressure is increased, it may lead to rupture or reflux of lymphatic channels and may cause chylous ascites or chylothorax (Kariya et al. 2019).

In terms of management of the chyluria symptoms, conservative treatment such as a high-protein and low-fat diet has a possibility to reduce chyluria. Other treatment options are sclerotherapy and embolization. Sclerosants induce an inflammatory reaction in the lymphatic vessels and blockade the communicating channels by fibrosis (Lovrec Krstić et al. 2021; Gurevich et al. 2018) presented three cases of chyluria and two of them underwent interstitial lymphatic embolization by percutaneously accessing the retroperitoneum lymphatic channels or retroperitoneal lymph node under fluoroscopic guidance. These two patients had chyluria due to increased thoracic duct pressure and they were completely resolved within 1 year and 7 months after the second embolization up to 1 year. One patient had improved symptoms with a low-fat diet. Nguyen et al. (2020) also used interstitial lymphatic embolization and accessed the lymphatic channel by puncturing the retroperitoneal lymph node through the abdominal wall and they put a balloon catheter in the TD in order to prevent reflux of embolic agent into the TD. A recent article by Hur et al. (2021) presented another approach into TD by retrograde through the left brachial vein or directly puncturing the TD followed by lymphatic embolization through a micro catheter. These treatments method for chyluria were based on disruption of the connection between renal calyces and lumbar interstitial lymphatic vessels.

Regarding to management of the causes, especially TD obstruction, there are only a few articles about the treatment methods. Thoracic ductoplasty with a balloon is reported to reduce lymphatic circulation pressure and restore the patency of the TD to treat chylothorax and chylous ascites (Kariya et al. 2019). The results showed that after 6 months, the symptoms did not recurrent. The use of stent in the management of TD obstruction has not been reported.

Therefore, stenting in management LVJ obstruction of the thoracic duct has not been reported and this article may be the first case reported in the literature regarding to treat TD occlusion. Until now, there has been one article describing TD stenting treatment for TD hypertension by Ghelfi et al. (2022). In this article, Ghelfi et al. reported two cases with cirrhosis and refractory chylous ascites for which transjugular intrahepatic portosystemic shunt. In these cases, portal hypertension increases lymphatic flow and may cause the pressure gradient without TD obstruction or stenosis. TD stenting may resolve the lymph-venous pressure gradient and chylous ascites. The stent used in our patient was a drug-eluting stent. A part of the stent was located in the subclavian vein aimed to keep the stent in the right location. The drug-eluting may prevent thrombosis.

Our patient had short-term success with balloon dilatation; however, re-obstruction appeared later. The cause of this condition may be fibrosis, intimal hyperplasia, or inflammation. In this case, the internal pressure of the thoracic duct was not measured before and after the procedure because of a lack of measuring tools. The cause of chyluria in this patient may be increased intrathoracic pressure leading to rupture or reflux of lymphatic channels into the urinary tract. The objective treatment in the present case was to release a blockage in the thoracic duct to reduce pressure in the peripheral lymphatic ducts. There is no blood reflux into the TD, possibly because the pressure in the subclavian vein is low and the same flow direction of the stent and the subclavian vein blood. Moreover, because we used drug-eluting stents, and there was no coagulation factor in the TD so anticoagulation was not used in this patient.

Thoracic duct stenting seems a feasible and practical approach in the treatment of LVJ obstruction. Follow-up is necessary to access the patency, location of the stent. Further studies are needed to confirm these results.

## Conclusion

Thoracic duct stent is a new procedure with successful approach in chyluria with thoracic duct stenosis or obstruction.

## Data Availability

All data are referenced from the medical records and stored in the hospital.

## Abbreviations

TD: Thoracic duct
LVJ: Lymphovenous junction
DCMRL: Dynamic contrast-enhanced magnetic resonance lymphangiography
CT: Computed tomography
